# Opportunities, challenges and trade-offs with decreasing avoidable food waste in the UK

**DOI:** 10.1177/0734242X20983427

**Published:** 2021-01-30

**Authors:** Shivalee Patel, Manoj Dora, John N Hahladakis, Eleni Iacovidou

**Affiliations:** 1 College of Health, Medicine and Life Sciences, Division of Environmental Sciences, Brunel University London, UK; 2 College of Business, Arts and Social Sciences, Brunel University London, UK; 3 Center for Sustainable Development, Qatar University, Qatar

**Keywords:** Food waste, surplus food, food supply chain, food donations, food redistribution, sustainability

## Abstract

Around 6 million tonnes of edible food are being wasted (post-farm gate) in the UK each year. This fraction of edible wasted food is known as avoidable food waste. In a circular economy food is a valuable resource that must be captured at all stages of the food supply chain and, where possible, redistributed for consumption. This can prevent avoidable food waste generation, and dissipation of food’s multidimensional value that spans environmental, economic, social, technical and political/organisational impacts. While the importance and benefits of surplus food redistribution have been well documented in the global literature, there are still barriers that prevent perfectly edible food from being wasted. This study looks at the main stages of the food supply chain, and amasses the opportunities, challenges and trade-offs associated with surplus food redistribution to the UK economy. It highlights points in the food system where interventions can be made, to improve food’s circularity and sustainability potential. Stakeholder interrelations, regulatory and socio-economic aspects are discussed in relation to their influence on decreasing avoidable food waste. The main output from this work is a diagrammatic depiction of where challenges and trade-offs occur along the food supply chain, and how policy and socio-economic reforms are needed to maximise avoidable food waste prevention, and the surplus avoidable food redistribution in the food supply chain for social benefit.

## Introduction

The circular economy (CE) concept entails a transformation of the way resources are used so that they can be retained in the economy for as long as possible. This concept has placed increased focus on the food sector, and particularly on food waste management (Iacovidou and Voulvoulis, 2018). According to the United Nations Food and Agriculture Organization (FAO) food waste accounts for one-third of all the food produced annually for global human consumption (FAO, 2013). There are two fundamental issues related to that: (a) the fact that almost 1 billion people suffer from food poverty; and (b) the profound negation of food’s embedded value (Facchini et al., 2018; Kummu et al., 2012). Embedded value may refer to greenhouse gas (GHG) emissions, chemical nutrients, fuels, energy and freshwater consumption associated with food production, processing, distribution, preparation and consumption, as well as the related social and economic value (Kummu et al., 2012). It may also refer to biodiversity loss due to land use change from forestry to agriculture, and associated impacts on natural, social and economic systems. When food is wasted, its embedded value is wasted too; for example, food waste contributes to approximately 3.3 billion tonnes of CO_2_e (excl. land use change), which accounts for around 8% of global GHG emissions (FAO, 2013).

On a European level the CE package^1^ and action plan (European Commission, 2015) and the European Green Deal (European Commission, 2019), and on a global level the Sustainable Development Goal (SDG) target 12.3, are increasingly promoting food waste prevention and reduction at all stages of the food supply chain (FSC). They posit that innovation and public awareness should pave the way to improving the sustainability of the food system and combating food fraud, while ensuring that food is redistributed back to the economy; alleviating poverty and meeting the CE principles. Redistribution is defined by the European Commission (2017) as ‘a process whereby surplus food that might otherwise be wasted is recovered, collected and provided to people, in particular to those in need’. It can occur via direct donations from donors to charities, or via food banks that store and distribute donated food to end users, for example charitable organisations (Hanssen et al., 2016). Food redistribution is considered to be an effective way of mitigating avoidable food waste generation and alleviating food poverty in local communities, including supporting small food producing businesses.

Nevertheless, food redistribution is not widely practised. This is contingent on the collaboration between different organisations that are directly involved in food production and handling, as well as organisations and individuals that are indirectly involved with the recovery of surplus food. Therefore, the absence of collaborations can severely hinder improvements in the effective redistribution of perfectly edible food. Previous studies on food and food waste management focused their investigation on identifying the potential of various techniques to improve the valorisation of food items to animal feed as a good management practice (Brancoli et al., 2017; Vandermeersch et al., 2014). Other studies tried to assess the environmental and economic benefits of food prevention initiatives in the retail sector (Albizzati et al., 2019; Martinez-Sanchez et al., 2016; Oldfield et al., 2016; Tonini et al., 2018).

Up until now, few attempts have been made in stressing the importance of collaboration between different stakeholders across the FSC, and in identifying the main challenges and opportunities related to food circularity and redistribution in the system. Studies have shown that current legislation and policies relevant to food redistribution and management can hamper the maximisation of food donations due to the inability of communities to adopt sharing practices that promote collective responsibility and trust within organisations (Bio by Deloitte, 2014; Morrow, 2019). Still, the opportunities and challenges of scaling up food redistribution and related trade-offs remain underexplored. Recognising this gap, this study aims to investigate the challenges, opportunities and trade-offs associated with food waste reduction and/or redistribution in the UK as a case study, to identify ways to support its effective recovery (as in terms of capturing for redistribution) and circularity in the food system. In its 25-year Environmental Plan, the UK Government set out a commitment to support the redistribution of edible food surplus from food businesses to individuals. Therefore, the purpose of this work is to report on progress in reducing avoidable food waste, and highlight where changes are mostly needed in the food system. It concludes by making recommendations for future actions that should be prioritised for promoting circularity in the FSC in the UK.

## Background

**Figure 1.**
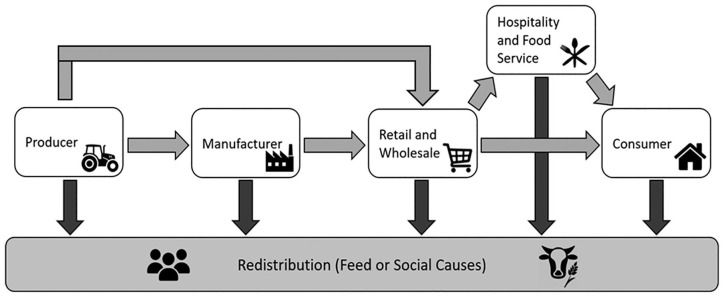
The main stages involved in the UK human FSC including a redistribution pathway. FSC: food supply chain. Source: reproduced from Defra (2017), Facchini et al. (2018) and Östergren et al. (2014).

Conceptually the food system comprises a set of processes that occur between the farm (production), fork (consumption) and end-of-life (EoL) management of food waste. The redistribution of food that is fit for purpose (i.e. for human consumption) to individuals, households and communities that experience food insecurity (Midgley, 2014), excludes the stages downstream of the food system that relate to post-consumer food waste collection and management. Therefore, our study focuses on the processes that occur between production and consumption of food, which involves all stages illustrated in Figure 1. This representation of the FSC provides a simplified view of the main processes involved in the upstream part of the food system (i.e. the FSC). It must be noted that the FSC is complex and includes also food packaging firms, producer cooperatives, certification and inspection organisations, food labs, advisors, traders and food service companies (Verdouw et al., 2016).

Understanding the way the FSC operates, makes it possible to identify barriers to food waste prevention, and opportunities for interventions that can promote improved food management practices. The term ‘food’ (or ‘foodstuff’) is commonly defined as:

any substance or product, whether processed, partially processed or unprocessed, intended to be, or reasonably expected to be ingested by humans. ‘Food’ includes drink, chewing gum and any substance, including water, intentionally incorporated into the food during its manufacture, preparation or treatment (European Parliament and Council, 2002).

This definition has been established by the European Commission (EC) of the European Parliament regulation on food law (European Parliament and Council, 2002) and does not include: animal feed; live animals, unless they are prepared for placing on the market for human consumption; plants prior to harvesting; medicinal products; tobacco and tobacco products; narcotic or psychotropic substances; and residues and contaminants.

A common definitional framework is required to: (a) establish comparable food waste estimates; (b) track the rate of food waste generation and prevention strategies reliably; and (c) to support policy-makers and stakeholders across the FSC. The EC funded project, *FUSIONS Definitional Framework for Food Waste* (Östergren et al., 2014), has reviewed over 300 peer-reviewed articles to develop a robust definitional system required for the formation of food waste prevention and management strategies. Table 1 contains key definitions established by the FUSIONS framework alongside other studies in the field.

**Table 1.** Definitions and sources of key terminology for addressing various types of food waste.

Term	Definition	Reference(s)
Food	‘*Food is any substance or product, whether processed, partially processed or unprocessed, intended to be, or reasonably expected to be consumed by humans. Food includes drink, chewing gum and any substance, including water, intentionally incorporated into food during its manufacture, preparation or treatment.*’	European Parliament and Council (2002) and Östergren et al. (2014)
	This definition excludes inedible parts of food; however, they are included in the FUSIONS technical framework	
Food waste	‘*Food waste is any food, and inedible parts of food, removed from the FSC to be recovered or disposed (including composted, crops ploughed in/not harvested, anaerobic digestion, bio-energy production, co-generation, incineration, disposal to sewer, landfill or discarded to sea).*’	Östergren et al. (2014), Parfitt et al. (2016) and FAO (2011)
	Food waste occurs in the latter stages of the FSC leading up to and including human consumption, i.e. wholesale, retail, HaFS and consumption	
Food loss(es)	Food loss refers to the decrease in edible food mass at the earlier stages of the FSC leading up to the preparation, transportation and display of food for human consumption, i.e. the production, post-harvest and processing stages	Parfitt et al. (2016) and FAO (2011)
Food surplus	Food surplus refers to the food produced beyond our nutritional needs and acts as a safeguard against unpredictable weather patterns affecting crops (however it has been highlighted by WRAP and FAO that the current state of global food surplus is threatening, not safeguarding, global food security)	Papargyropoulou et al. (2014) and Parfitt et al. (2016)
Theoretically avoidable	Food waste that could in theory be edible with or without further processing; includes only the portion of food waste that was intended for consumption (e.g. ingredients or product lost during changeover or cleaning, quality assurance rejects, etc.)	Parfitt et al. (2016)
Practically avoidable	Food waste that is edible and can genuinely be prevented (e.g. during the manufacture of flavoured milk drinks some product waste will occur during line cleaning between batches; although the milk is theoretically avoidable and edible, it is not practically avoidable)	Parfitt et al. (2016)
Unavoidable	Food that is not, or has never been, edible under normal conditions (e.g. shells, fruit and vegetable peelings, coffee grounds or bones)	Parfitt et al. (2016)

In this study we use the term ‘avoidable food waste’ to refer to both theoretically avoidable food waste and practically avoidable food waste. In addition, food surplus and surplus (avoidable) food waste are used interchangeably, as we consider that what is avoidable can be redistributed back to the system as surplus food. This also points to the fact that the definition of surplus food waste is ambiguous (with some surplus food products being unavoidably wasted in the FSC), and it is considered by the industry as a non-standard category (Alexander and Smaje, 2008). We acknowledge that the use of avoidable food waste/food surplus in this study may be an oversimplification; yet, uncertainty related to existing data on avoidable, unavoidable and surplus food waste generation makes it difficult to robustly distinguish food arising from each of these categories.

## Methodology

Focusing on the UK as a case study, we carried out a scoping literature review to address the following research questions: (1) What are the key organisational challenges?^2^ (2) What opportunities^3^ exist for maximising surplus food redistribution? (3) What are the associated trade-offs?^4^ Scoping reviews can support the ‘mapping’ of existing literature, synthesise research evidence to provide an in-depth representation of the current situation (Okoli, 2015; Okoli and Schabram, 2010; Popay et al., 2006), and identify gaps for future research (Venkatesh et al., 2007). They are often called ‘mapping reviews’ (Anderson et al., 2008).

The scoping literature review was performed using the literature databases Scopus, Web of Science and Google Scholar. To query articles relevant to our research questions we used the keywords: ‘edible food waste’ OR ‘avoidable food waste’ OR ‘surplus food’, ‘UK’ OR ‘Europe’, ‘food losses’ OR ‘food waste’, ‘food redistribution’, ‘food waste prevention’, ‘food waste policy’ AND ‘sustainable food management’. It is important to note that the latter terms are often used interchangeably with terms such as ‘food sharing’, ‘food prevention strategies’, ‘food charities’ and ‘food poverty alleviation’, which have also been included in the review.

Additional searches were carried out where necessary and relevant to further decipher specific aspects of interest. For example, governmental documents published by the Department for Environment, Food and Rural Affairs (Defra) and reports published by the Waste and Resources Action Programme (WRAP) were used so long as the sources contained strict or meaningful bibliographic control. Furthermore, policies such as EU Directives, national and international laws were referred to during data analysis. The official websites of surplus food redistribution initiatives have been used to collect information, which was used to critically evaluate the impeding challenges posed by current legislation and management practices, alongside behaviour and relationships among stakeholders (and their influences) (Sterman, 2000), and to outline potential opportunities and associated trade-offs.

The retrieved literature was scrutinised and analysed using the CVORR framework. CVORR stands for Complex Value Optimisation for Resource Recovery; it is a system-of-systems approach developed for assessing and evaluating multidimensional value dispersal (capture, dissipation and possibly creation) across the natural and man-made resources production-consumption-management processes; identifying where interventions are needed in such systems (Iacovidou et al., 2017). The CVORR baseline analysis includes the following steps: (1) definition of goals and scope; (2) definition of system boundaries; (3) identification of system processes and quantification of mass flows; (4) identification and quantification of monetary flows and stakeholder identification; and (5) analysis of system structure, dynamics and drivers (Iacovidou et al., 2020). The scope of the present study is to analyse the challenges, opportunities and trade-offs related to surplus food redistribution (step 1), in the UK (step 2). A food mass flow analysis is available in Facchini et al. (2018); here we provide an insight into the avoidable food waste produced that could be distributed as food surplus in the FSC (step 3). Although the mapping of monetary flows was excluded due to the complexity of the FSC combined with time limitations, the stakeholders involved in the FSC were identified (step 4). Then we placed emphasis on the system structure and drivers in order to finalise our analysis and make it relevant to decision- and policy-making (step 5). We employed CVORR to get an overview of the avoidable food waste management in the UK and to address the three research questions outlined above.

## Results

### Avoidable food waste in the UK

In the UK, the FSC involves the structures and processes responsible for providing access to food to the UK population. Understanding the way that the FSC functions is particularly important in understanding the relationship between the different stakeholders involved, as well as of their role in supporting or hindering surplus food redistribution (Parfitt et al., 2010). Primary food production is a complex process that encompasses many activities, for example livestock rearing, fishing and farming, that lead to the production of agricultural products. A considerable proportion of these products is transformed during the manufacturing stage into other forms of food products, which are then transported to wholesale and retail points in the FSC. The rest of the fresh produce is directly entering the retail and wholesale stage (Figure 1). The heterogeneous nature of primary food production makes the quantification of avoidable food waste difficult to accurately measure, and as a result food waste quantification in the UK usually begins at post-farm gate (Stenmarck et al., 2016; WRAP, 2018). Nonetheless, it is worth mentioning that 30% of vegetable and fruit crops in UK farms can remain unharvested, contributing to a staggering 2.5 million metric tonnes (Mt) of *pre-farm gate* avoidable food waste (Stuart, 2009; Vision 2020, 2013).

In 2018, the total amount of food waste generated in the UK *post-farm gate* was around 9.5 Mt (Facchini et al., 2018; WRAP, 2020b). Household food waste accounted for 6.6 Mt (WRAP, 2020b) of the total food waste generated in the UK (post-farm gate), 0.4 Mt less than the 7.1 Mt reported in 2015 (WRAP, 2018), making up 70% of the total UK food waste production. Over two-thirds of this waste (68%, which equates to 4.5 Mt) was avoidable (i.e. that could have been eaten), with a value of almost £14 billion (based on 2018 monetary values).

**Figure 2.**
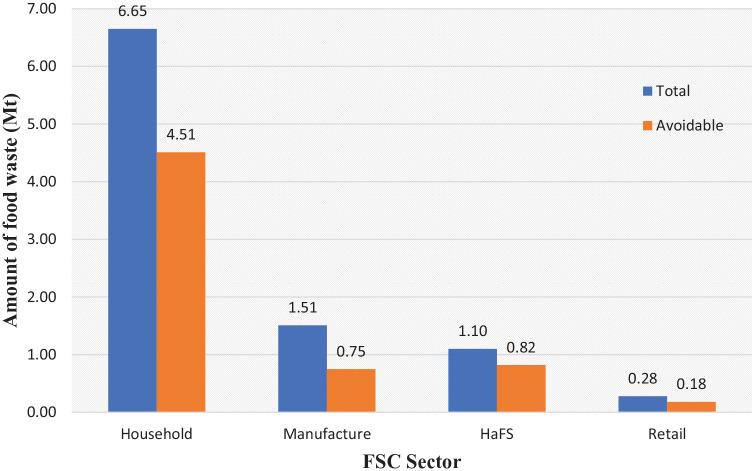
Total and avoidable food waste generated by sectors of the FSC and by households in the UK. FSC: food supply chain.

The remaining 30% (2.9 Mt) of the food waste produced in the UK (post-farm gate) originated from the manufacture, retail, and hospitality and food service (HaFS) sectors, contributing approximately 1.7 Mt, 0.26 Mt and 1 Mt of food waste, respectively. Over two-thirds of this waste (65%, which equates to 1.9 Mt) was edible food that could have been salvaged, with a value of over £5 billion (based on 2018 monetary values) (WRAP, 2020b). Specifically, in the HaFS sector^5^ 75% of the food waste generated (i.e. 0.75 Mt) could have been avoided, whereas in the manufacture sector a staggering 50% of the food waste produced could possibly be avoided (i.e. 0.8 Mt). In the retail sector, lack of data makes it hard to predict how much of the food waste produced could have been avoidable (although it can be assumed that the vast majority of food waste in this sector is avoidable either theoretically and practically) and therefore we used the FSC average (i.e. 65%). Figure 2 presents the amount of avoidable food waste generated against total food distributed/consumed in the FSC and household.

It must be noted that data reported on avoidable food waste generated in the manufacture and HaFS sectors can be associated with a degree of uncertainty as accounting methods vary (Alexander and Smaje, 2008). For example, some data could relate to both the HaFS and manufacture sectors, or to manufacture and retail sectors, creating confusion and preventing robust estimates. Around 0.13 Mt of food waste generated in the HaFS sector is ready-to-serve food items and meals produced by the manufacturing industry, and it remains unclear how these are included in the wastage figures (WRAP, 2013).

In June 2012, the UK Government launched the HaFS agreement to prevent food waste (and associated packaging waste) by 5%, while increasing recycling rates up to 70% through collaborative sector action (WRAP, 2013; WRAP, 2020b). This was in addition to the Courtauld voluntary agreement that was launched in 2005 to create solutions and technologies to minimise food and primary packaging waste; divided into three distinct phases (known as Courtauld 1, 2 and 3). In 2015 the HaFS and the Courtauld (2005) agreements were brought under a new agreement known as the Courtauld Commitment 2025 (WRAP, 2020a).

Courtauld 2025 (or C2025) is an ambitious voluntary agreement that aims to bring together organisations across the entire FSC to cut down food and drink waste (and the carbon, water and waste associated with it) to one-fifth over a period of 10 years, and promote sustainable food and drink production and consumption. Achieving this commitment requires a change in the ways that governments, individual companies or community groups operate, which can be supported by the creation of powerful partnerships between organisations that would not normally work towards common goals (WRAP, 2020).

Prevention of food waste at source, surplus food redistribution and diversion of surplus food into animal feed are all needed to meet the UN Sustainable Development Goal target 12.3 and achieve the C2025 target. Yet avoidable food waste is still being generated in the UK FSC; amounting to 6.4 Mt of avoidable food waste (post-farm gate) in the UK. WRAP (2018) reports that around 55 kt of food surplus was redistributed in 2018, and that there is potential to increase this by an additional 190 kt from the retail and manufacturing sectors (approximately 80 kt from retail and 110 kt from manufacturing), as well as from other parts of the FSC (e.g. primary production and HaFS) (WRAP, 2018). Therefore, there remains the need to increase the amount of food surplus redistributed significantly, and reduce the amount of avoidable food being wasted. That said, all stakeholders involved in the FSC need to work collaboratively to identify ways of increasing the redistribution of surplus food.

### Challenges and trade-offs to avoidable food waste reduction

#### Regulatory challenges and trade-offs

At the time of writing, the UK adhered to the European legislation for food safety, hygiene, consumer information and management, including the EU Regulation 852/2004 on the hygiene of foodstuffs (to ensure a high level protection of human life and health); EU Regulation 1169/2011 on the provision of food information to consumers; and EU Regulation 178/2002 laying down the general principles and requirements of food law. These regulations lay down the rules for food safety and hygiene and attribute FSC operators the same responsibility for both the food they placed on the market, and the food they donate to charities for redistribution, with the latter adhering to EU legislation concerning traceability (Canali et al., 2017).

Different food types come under specific regulations to protect the retailer and consumer, with the trade-off of contributing to potentially avoidable food waste generation. For example, strawberries fall under the Specific Marketing Standards in EU Regulation 543/2011 that require as a minimum that produce must be intact, undamaged, sound, clean, practically free from pests and pest damage, free of abnormal external moisture, and free of any foreign smell and/or taste; the regulation also includes specifications for shape, size and colour (WRAP, 2016). Traders – individuals or bodies that display, offer for sale, sell or market (including distance selling, online or otherwise) produce in any way either within the EU, for export outside the EU or for import into the EU – that act as intermediaries between primary food producers and manufacturers, wholesalers and retailers have the responsibility to abide by these regulations (European Commission, 2020). They often adopt additional stringent rules for product quality standards to ensure they secure the right selling prices and keep their clientele happy.

While regulations ensure food safety and product liability from production to consumption (Bio by Deloitte, 2014; Morrow, 2019), there is no flexibility in the rules to facilitate surplus food redistribution (Bio by Deloitte, 2014; De Boeck et al., 2018), which makes any surplus food donation by the FSC stakeholders difficult (European Parliament and Council, 2002). In addition, there is a lack of EU food regulations that are specifically designed for surplus food redistribution. This makes FSC stakeholders reluctant to donate their food surplus, to avoid the risk of being legally pursued in case food-related health problems occur that may harm their reputation (Canali et al., 2017). This, in turn, creates a barrier in regards to enabling surplus food redistribution initiatives.

FSC stakeholders with food surplus are often inclined to discard it in order to avoid dealing with liability risks (De Boeck et al., 2018). Circumventing such obstacles can be achieved via social and financial investments that support the development of the infrastructure needed to carry out such activities (e.g. hiring staff to complete adequate safety and hygiene checks, tracking and archiving information regarding food status, etc.), such as in France (Mourad, 2016). In return for complying with the law, surplus food donors may receive a tax credit equal to 60% of the surplus donated food value to a limit of 0.5% of company revenue subject to corporate income tax (Bio by Deloitte, 2014). While fiscal instruments like this can successfully increase surplus food donation volumes, their compatibility with the EU VAT Directive (that makes definitions such as ‘abandoning’ or ‘exempting’ VAT liability ambiguous) can create loopholes and potential fraudulence in the system.

Additional trade-offs associated with legislative aspects include the use of terms, such as ‘when it’s necessary’, ‘if necessary’ and ‘if applicable’ (as in EU Regulation 852/2004 on the hygiene of foodstuffs), which are frequently misinterpreted by businesses creating uncertainty and deterring redistribution efforts (Bio by Deloitte, 2014; De Boeck et al., 2018). The provision of food information to consumers (as in EU Regulation 1169/2011 on the provision of food information to consumers) states that the ‘Best before’ or ‘Use by’ dates must be determined by the food business operator based on the composition of a product. The ‘Use by’ date on food is about safety, which means that food cannot be eaten beyond that date; thus, food items with the ‘use by’ must be discarded (unavoidable food waste) beyond the listed date and cannot be donated (FAO, 2013; FSA, 2020). The ‘Best before’ date is about quality (FAO, 2013; FSA, 2020). Food items beyond their ‘Best before’ date that appear to be in an acceptable condition, may still be safe for consumption and can be donated provided that they continue to be stored properly (Bio by Deloitte, 2014; Parfitt et al., 2016). Some FSC stakeholders may be unaware that foods exceeding the ‘Best before’ date remain edible (Bio by Deloitte, 2014; De Boeck et al., 2018; European Commission, 2017), and that legislation does not prohibit their redistribution given that it is safe to do so (as in EU Regulation 178/2002 laying down the general principles and requirements of food law). However, the perceived food quality of products past their ‘Best before’ date does not always imply food safety. For example, a food product may appear of high quality but could be contaminated with undetected pathogenic organisms, toxic artificial chemicals or physical hazards) (Aung and Chang, 2014; Morrow, 2019).

Additional barriers to surplus food redistribution include: proximity, which can hinder donations, especially with fresh foods (e.g. fresh fruits and vegetables and ready-to-eat composite products) that have a short-shelf life (Bio by Deloitte, 2014); distribution of cooled or frozen food (De Boeck et al., 2018); lack of structure, organisation and knowledge on food hygiene by volunteers; and financial and administrative burdens incurred by donors (De Boeck et al., 2018).

#### Challenges related to FSC stakeholder dynamics

The stakeholders involved in the FSC and their relationships play an important role in the way food is distributed, stocked and wasted. Primary food producers rely heavily on manufacturers and wholesalers/retailers for selling their produce. For example, small-scale farmers and fishermen rely heavily on wholesalers/retailers for selling their fresh produce (e.g. vegetables, fruits, fish, eggs), while large-scale farmers often rely on manufacturers for selling their crops, meat, fish and other produce. For small-scale farmers, alternative sales routes in secondary markets (e.g. selling strawberries to manufacturers for yogurts, juice, jam production) are not particularly attractive due to the lower financial incentives accrued by such exchanges. For example, fresh fruits (e.g. strawberries) and vegetables fetch a better price if sold as fresh fruit in the primary market. If it doesn’t meet the specifications set by the traders, wholesalers and retailers they could be sold to the processing industry, but this market is very small in comparison to the fresh market (WRAP, 2016). As a result, small-scale farmers often find it sensible to store their produce with the aspiration to sell it to wholesalers/retailers and fetch a better price, which creates a time lag that leads to edible food being spoiled. Unexpected changes, as for example cancelled orders by the wholesalers and retailers, can also lead to the generation of avoidable food waste, as well as failure to meeting product specifications set by the traders, manufacturers, wholesalers and retailers (Parfitt et al., 2016; WRAP, 2016). Table 2 presents the causes and drivers of avoidable food waste generation, and the key stakeholders that impact and are impacted by this spoilage.

**Table 2.** Causes and drivers of avoidable food waste production which occur or originate from the UK processing/manufacturing sector.

Subsector	Causes of surplus food production	Stakeholders impacted
Fruit and vegetables (loose and packaged)	Strict product specifications	Farmers; importers; traders; manufacturers; package/label designers; wholesalers; retailers
	Mishandling and improper conditions of storage (bruises and other damage)	
	Difficulty in forecasting volumes of supply and demand (overproduction)	
	Seasonal variations resulting in higher than expected crop yields	
	Temperature control failures during transportation	
	Market volatility impact on stock	
	Package/labels used other brand/aesthetic issues (attractiveness to consumers)	
	Package size not preferred by buyers/consumers	
Meat, poultry and fish (fresh)	Strict product specifications	Farmers; importers; traders; manufacturers; package/label designers; wholesalers; retailers
	Animal by-product safety regulations – labelling that shortens their shelf-life	
	Seasonal variations and holidays/special events (e.g. Christmas, summer, bank holidays)	
	Temperature control failures during transportation	
	Mishandling and improper conditions of storage	
	Market volatility which affects price and consumer preference	
	Package/labels used that prolong shelf-life (freshness) and aesthetic quality	
Bakery goods and breakfast cereals	Product specification	Manufacturers; package/label designers; importers; traders; wholesalers; retailers
	Over-baking or not baking items to aesthetically satisfactory levels	
	Fragile products with variable shelf-life (1 day–6 months)	
	Bulk purchasing ingredients that pass shelf life	
	Unexpected delisting of products by retailers	
	Package/labels used	
Soft drinks/fruit juices	Overproduction	Producers; manufacturers; package/label designers; importers; traders; wholesalers; retailers
	End of retail promotional deals	
	Defects on packages	
	Labels used and other brand/aesthetic issues (attractiveness to consumers)	
	Package size not preferred by buyers/consumers	
Pre-prepared meals	Missing ingredients caused by human error leads to product destruction (e.g. pizza toppings)	Producers; manufacturers; package/label designers; importers; traders; wholesalers; retailers
	Over-ordering of ingredients because of minimum order volumes not used in time	
	Mishandling and improper conditions of storage	
	Packaging/labelling mistakes (e.g. wrong date coding) and changes by retailers	

Source: reproduced from Parfitt et al. (2016) and Mena et al. (2011).

Owing to the strict product quality standards and other specifications and cosmetic standards set by retailers and driven by perceived consumer demands, 30% of vegetable and fruit crops in UK farms can remain unharvested (Stuart, 2009; Vision 2020, 2013). Yet, the inherent characteristics of food such as its size, shape, texture and maturity, especially of fruits and vegetables, mean that the strict quality standards can be a barrier to their harvest and sale on the market. For example, berry size must be above 18 mm to pass EU standards but over 25 mm to pass most retailer specification (WRAP, 2016), whereas over 9% of mature strawberry crops are wasted (i.e. 10 kt), worth £24m. Moreover, 19% of all lettuces growing in the UK were unharvested (i.e. 38 kt), worth an estimated £7m (WRAP, 2016). Other causes of avoidable food waste at the primary production stage can be the lack of adequate harvest and control systems and technologies used (e.g. automated harvesting, trawl fishing and use of non-selective gear catches fishes that are not consumed; industrial livestock farming causes stress to animals and consequent death) (Canali et al., 2017; House of Lords, 2014), as well as the shortage of EU labour post Brexit, weather-related impacts on crops (e.g. strawberries and lettuce), pest damages, overproduction and price volatility (Table 2). In regard to the latter, food prices are subjected to market volatility and when the price of food drops, farmers would rather leave the crop unharvested as it would cost more to harvest it. This volatility is largely dependent on the retailers that often seek out the cheapest produce, tighten their cosmetic specifications and continue to import the cheapest produce from overseas (Vision 2020, 2013).

At the processing/manufacturing sector, where raw food materials are turned into products for intermediate or final consumption, there is an increased reliance between producers/manufactures and raw food suppliers, package and label designers/suppliers, and other ingredient suppliers at one end, and retailers/wholesalers or other food manufacturers who are the main buyers of the food products manufactured at the other. Of these relationships, the manufacturer-retailer is the most important as it determines and controls the types and amounts of food products placed on the market. The large number of manufacturers and retailers has resulted in a vast heterogeneity and multiplicity of food products, which are manufactured under different quality specifications often determined by each manufacturer and/or retailer. For example, the ingredients used, the texture and taste of the end food product, its smell and appearance, the declaration of allergens, as well as the type, design, durability and functionality of food package and labels used, can vary considerably from one factory/retailer to another. These decisions involve many stakeholders often with competing interests and values, which affect indirectly the way product specifications set by the retailers for both the food and package design and type are met, and in turn, may directly impact on food purchasability and durability (shelf-life). In addition to the range and nature of food products, the type, efficiency and advancement of technologies used (e.g. mechanical peeling and handling of fruits and vegetables) and associated damages and failures (Canali et al., 2017), and the quality management control measures put in place at the manufacturing stage (e.g. operation standards, optimal storage and handling) are additional factors that can contribute to the generation of large amounts of avoidable food waste by any stakeholders involved in this stage (Swaffield et al., 2018).

Avoidable food waste generation can also occur during the transport of food along the supply chain, due to inappropriate storage and handling, especially for fresh products. For example, packaging defects can lead to broken and damaged food items, while inappropriate use of packaging (e.g. size, material and type) and labelling (e.g. packaging mismarked and mislabelled) that may lead to incorrect inventory and shelving, may also give rise to avoidable food waste (Canali et al., 2017) (see Table 2).

In the wholesale/retail sector there are several factors at play that can lead to the production of avoidable food waste, which depend on the relationships that retailers establish with manufacturers, producers and quality control managers. In regards to the latter, storage conditions, fridge/freezer errors and inappropriate use, lack of organisational controls and quality checks at product stocking/shelving, and seasonal irregularities can result to large amounts of avoidable food waste. Moreover, contracts and agreements for deliveries and management of unsold products, for example ‘take-back agreements’, can lead to surplus food being returned to the suppliers, at zero cost for the retailers (Ghosh and Eriksson, 2019). Rather than redistributing food surplus to people in need, retailers often opt to utilise the ‘take-back agreements’ and avoid the responsibility of dealing with surplus food management. This results in avoidable food wastage earlier up in the FSC; transferring the problem from the retail stage to the supply/manufacturing stage. Furthermore, with such take-back schemes, wholesalers and retailers have a low incentive to accurately forecast supply and demand fluctuations, which can lead to surplus avoidable food waste left to be disposed of by the weaker actors (Ghosh and Eriksson, 2019; Stenmarck et al., 2016). Additional challenges to surplus food redistribution include: lack of structure, organisation and knowledge on food hygiene/safety; and financial and administrative aspects.

Notwithstanding the implications of the above relationships, at the retail stage the most important relationship is that between retailers and consumers. The efforts of retailers to supply a range of products to their customers in an increasingly competitive market is one of the reasons for food surplus being generated. For example, promotions or discounts in competing stores, aesthetic quality standards (consumer driven), damaged or incorrectly packaged products due to manufacturing errors and/or distribution and storage incidents, product mislabelling (Midgley, 2014), shelf life, and number of customer visits (Vågsholm et al., 2020), seasonal ordering, over-ordering, and new product testing or developments, unpredictable events such as sharp weather changes (Parfitt et al., 2016), and poor quality control add to the volume of avoidable food waste generated (Alexander and Smaje, 2008; Facchini et al., 2019). Market volatility and time-dependence that urges retailers to supply products to satisfy customer demands may also lead to over-supply which results in avoidable food waste generation especially when it involves perishable food (Alexander and Smaje, 2008; Vågsholm et al., 2020). The interpretation of ‘Use by’ or ‘Best before’ date by both the retail employees and consumers is another challenge that leads to avoidable food waste generation in the wholesale/retail sector (Canali et al., 2017; Facchini et al., 2018; Ghosh et al., 2016), as explained above. This creates tension between consumers and retailers. Retailers wish to extract profit from items up to the moment they are unusable, and hence minimise the amount of food products that go to waste. For consumers value is maximised when they pay for food that is perceived to be of high quality (Vågsholm et al., 2020).

In the HaFS sector the most important relationship is again that between service providers (e.g. staff catering, quick service restaurants (QSRs) and fast food, restaurants, pubs, hotels and leisure) and customers (i.e. consumers). The food surplus generated at this stage could be related to the overproduction of meals and unwanted food due to customers’ preferences and mistakes occurring during ordering (WRAP, 2013). Personal preferences may be related to food and drink not eaten due to allergies and/or other health reasons, or simply to not wanting to eat a particular food or part of a food item (WRAP, 2018). Personal preference was suggested to be the third largest reason for avoidable food waste accumulation (roughly 14%) (WRAP, 2018). Over 20% of restaurant, pub, services and leisure food is wasted out of the total volume of food purchased; this is approximately one in five potential meals. Subsectors such as QSRs and staff catering, which serve lighter meals and/or snacks and ready-to-eat foods, tend to dispose of one in every six potential meals. The top three causes of food waste within the HaFS sector arise from spoilage (21%), food preparation (45%) and consumer plates (34%) (WRAP, 2013). The quantity of waste produced by the HaFS sector is influenced by on-site food preparation, overproduction of meals, menu choice and extent to which consumers leave food unconsumed (WRAP, 2013).

Finally, we have consumers; the most important stakeholder in the food value chain. The largest amount of avoidable food waste is produced in UK households. A complex factor contributing to food wastage is consumers’ behavioural patterns and eating habits. Besides, some key organisational aspects at the household level may also need to be taken into account as they can affect avoidable food waste generation rate. These aspects can be associated with food purchasing and preparation practices, storage conditions and the use of suitable technologies, unplanned and spontaneous shopping and meal preparation, attraction to promotional offers or new products, as well as excessive meals preparation that consumers may not be able to eat (Canali et al., 2017; Facchini et al., 2018). WRAP (2018) found that the largest contributor to household avoidable food waste generation was food not being consumed in time, or perceived so due to the misunderstanding surrounding the ‘Best before’ date on products (WRAP, 2018; House of Lords, 2014; WRAP, 2008). Personal preference and eating habits was found to be the second largest contributor to avoidable food waste generation (WRAP, 2018).

Seasonal variations and special events (e.g. Christmas, Easter and other religious celebrations, bank holidays) are another challenge in tackling avoidable food waste in households, where consumers tend to deviate from ordinary routines, and buy and/or prepare more food than necessary (Canali et al., 2017). Additional factors that may lead to avoidable food waste generation include: food received as a gift; food bought for parties/guest visits; purchase of new food; frequency of shopping; frequency of dining outside the household; and bulk shopping (Canali et al., 2017). Studies reported that foods that are frequently disposed of are fresh vegetables and salads, drinks, bakery goods, home-made and pre-prepared meals, and dairy and eggs, and their amounts fluctuate depending on the proportion of food purchased and/or consumed outside the home (Defra, 2017; WRAP, 2017). Moreover, economic factors, such as household incomes and food prices, have been found to have an impact on avoidable food waste generation and purchasing behaviour; for example, rising food prices reduces consumer purchasing and food waste although overall spending and food sale revenue remains unaffected (Britton et al., 2014).

### Opportunities and trade-offs associated with avoidable food waste reduction

In the UK, there are opportunities for promoting the prevention and recovery of surplus avoidable food waste, for example via national and local initiatives, physical and virtual platforms, and via consumer engagement using electronic applications. A crude categorisation of opportunities for surplus food redistribution in the UK is presented in Table 3.

**Table 3.** Opportunities for avoidable food waste reduction via surplus food redistribution in the UK and its potential trade-offs.

Category	Description of activities	References
HaFS Initiatives	Restaurants and quick-service restaurants (QSRs) initiate their own schemes in an effort to distribute unsold food products to people in need, via charities and local community groups that claim it and collect it	KFC (2019) and WRAP (2019)
	*Example initiatives: KFC’s ‘Food Donation Scheme’*	
Physical platforms	Established by non-profit organisations that connect FSC stakeholders (e.g. processors/manufacturers, wholesalers/retailers and traders, hotels, restaurants, caterers) to charities and community group members that help homeless people and others with no, or low incomes, and with poor access to nutritious food, to gain access to fresh and dry food, or prepared nutritious meals	City Harvest (2020), FareShare (2020), FoodCloud (2020), UKHarvest (2020), The Felix Project (2020), FoodCycle (2017), Plan Zheroes (2020), and Olio (2020)
	*Examples: City Harvest (local); FareShare (nationwide); FoodCloud Hubs (local); FoodCycle (nationwide); Olio—Food Waste Hero Programme (nationwide); Plan Zheroes (local, markets only); The Felix Project (local); UK Harvest (local)*	
Online platforms	Established by non-profit organisations to connect FSC businesses in the production, processing/manufacture, wholesale/retail and HaFS sectors to post online descriptions of food that they cannot sell but are still edible and adhere to food safety regulations, and for nearby charities and local communities to claim that food and collect it for distribution to people in need	FareShare (2020), FoodCloud (2020) and Plan Zheroes (2020)
	*Examples: Plan Zheroes (local); FareShare Go (nationwide, operated by FoodCloud)*	
Food sharing applications	Free mobile applications that connect HaFS sector and individuals to other individuals that are in close proximity and seek to exchange food for free, or purchase food at lower prices	Too Good To Go (2020), Olio (2020) and Karma (2020)
	*Examples: Olio; Karma; Too Good to Go*	

FSC: food supply chain.

Currently, in the HaFS, there are not many initiatives. Stakeholders in this sector are already connected to non-profit organisations that collect their food surplus. One example initiative is promoted by Kentucky Fried Chicken (KFC) UK; the QSR chain would typically send all its unsold food to be treated for energy recovery. However, with increased awareness over the importance of finding alternative uses to food that is perfectly edible (hence reducing surplus avoidable food waste) and the increased number of people that are in need of food, the company’s priorities have changed and ‘feeding people first’ has become its goal (KFC, 2019; WRAP, 2019). An important trade-off resulting from the distribution of this food surplus is the lack of nutritional benefits, and potential harm to health when it is consumed by the same people on a relatively frequent basis.

Physical methods of utilising food surplus in the UK are practised by several stakeholders (i.e. non-profit organisations), whose activities are differentiated by the types of food they accept and with which stakeholders in the FSC they connect. For example, FoodCycle and The Felix Project do not accept raw meat/fish, while all non-profit organisations do not accept food past its ‘Use by’ date and food that has already been cooked or prepared. Almost all stakeholders work with all segments of the FSC to source food surplus, except Plan Zheroes Collection programme that sources food from local markets (London) and FoodCycle that accepts food from wholesalers/retailers and markets operating at national level. The biggest non-profit organisation sourcing food surplus in the UK is FareShare.

FareShare consists of 21 regional centres across the UK (five of which are managed directly by FareShare – the rest are managed by third-party independent charities in partnership with FareShare), which accept food from different points in the FSC and deliver it to charities and community groups that turn it into nutritious healthy meals for people in need. It also supports local charities directly by connecting them with retailers (e.g. Tesco, Waitrose, Asda) via the FareShare Go electronic application. Charities and organisations such as the Trussell Trust – a network of over 1200 food banks operating across the UK providing non-perishable food to vulnerable people and people in need via regular food donations and vouchers that entitle them to three days’ worth of nutritionally balanced foods – can gain access to both perishable and non-perishable food surplus that is fit for human consumption (FareShare, 2020).

FareShare operatives adhere to all relevant food safety legislation including: Food Safety Act 1990; Food Hygiene Regulations England/Scotland 2006; and Regulation EC852/2004 Hygiene of Food Stuffs, ensuring the safety of food delivered to end-users. Some food donors deliver the food directly to FareShare warehouses, or FareShare operatives visit wholesale/retail outlets and collect food surplus on an ad hoc basis (Alexander and Smaje, 2008). During the collection stage, operatives can either accept or reject food if it is potentially unfit for human consumption. Additional food surplus may be rejected at the depot if this is judged to be unfit for human consumption (packaging is also removed from food items) (Alexander and Smaje, 2008), and the truly avoidable food waste is prevented by being transformed into healthy meals (perishable) or prepared for distribution to people in need (non-perishable) (FareShare, 2020). This encourages businesses to donate foods without risking negative brand image (Bio by Deloitte, 2014; De Boeck et al., 2018). Via this transaction route donors and food banks can develop better relationships that enable higher recovery of surplus food (Bio by Deloitte, 2014).

There are several trade-offs associated with the use of this model: (a) perceived impact on food donors when it comes to the type/amount/quality of food donated and their reputation (e.g. small donation of unsold sandwiches from a single retailer, or freshness, condition and quality of retailer brand items that may impact on their reputation) (Alexander and Smaje, 2008); (b) impact on food recipients’ dignity (Cooper et al., 2014) and loss of cultural preferences and personal tastes (Thompson et al., 2018); (c) lack of control over the types of food surplus provided to charities and community groups; (d) infrequent availability of food surplus which increases the vulnerability of charities/community groups that are increasingly reliant on this food stream; (e) shift of food ownership from other FSC stakeholders to the non-profit organisations that accept their food products, which (non-profit organisations) are then liable for food rejects/waste disposal; and (f) food rejected at source reported as donated, hence not being properly accounted as waste (Alexander and Smaje, 2008). This serves the interests of both retailers and manufacturers as it places the accountability for waste minimisation elsewhere in the system (from FSC donors to third party organisations) (Alexander and Smaje, 2008), or nowhere at all (when logistics do not reflect true amounts) creating discrepancies between reported waste and actual amount produced.

The Foodsharing.de initiative operating in many European countries (e.g. Germany, Austria) has dealt with these issues by introducing a food-rescue network made of various community-managed resources such as food fridges, and an online platform. The public fridges are open-access to everyone and the food inside is owned by no individual or organisation (Morrow, 2019). This lowers the barriers for people to donate food, and reduces the stigma associated with accepting aid; hence safeguarding the sense of dignity and respect for the users (Morrow, 2019). This initiative promotes practices that increase collective responsibility and trust within society; they assist in alleviating food poverty in society while reducing avoidable food waste (Morrow, 2019; Schanes and Stagl, 2019).

Online platforms that support surplus food redistribution such as Plan Zheroes and FareShare Go encourage relationships between food businesses and charities by simplifying the donation process using technology applications, such as interactive online maps (Plan Zheroes, 2020). Via the online maps FSC businesses can easily find and connect with charities and local community groups that are signed up to the platform and are able to receive food surplus, which is then converted into nutritious meals (FoodCloud, 2020; Plan Zheroes, 2020). Charities and community groups are responsible for the collection of surplus food from the business, which can often be a trade-off as long distances create an important time lag for perishable fresh foods (e.g. fresh fruits and vegetables and ready-to-eat composite products) (Bio by Deloitte, 2014). Lack of cooled or frozen storage can be a limitation for food banks to hold large donations of fresh foods potentially leading to avoidable food waste (De Boeck et al., 2018). Moreover, the lack of structure, organisation and knowledge on food hygiene by the food bank volunteers can be a deterrent for retailers to donate food to protect their brand image in the case of an incident (De Boeck et al., 2018).

At the HaFS and household stages of the FSC opportunities for avoidable food waste prevention and surplus food redistribution can be practised via mobile applications. Olio (UK) connect individuals and businesses to share and receive surplus food locally (Olio, 2020). Approximately 50% of surplus food posted on Olio is relocated within an hour, which is beneficial for short shelf-life products (WRAP, 2019). Moreover, between 70% and 90% of food and drink products added to the Olio app is successfully redistributed (WRAP, 2019). Sources can include food reaching the end of its marketable life, unused household products or HaFS surplus. Users simply upload an image to the app with a description of the food item(s) and details of the place and time of exchange (Olio, 2020). The Karma and the Too Good To Go apps connect HaFS businesses that sell their leftover products at low prices with individuals that go and pick them up (Karma, 2020; Too Good To Go, 2020).

There are a number of potential trade-offs with the use of such technologies. For example, the lack of public awareness in regards to what is considered to be safe to consume is very subjective and may cause dissatisfaction with the use of the app. Aside from personal preferences, there is also the issue of food safety and hygiene; not all people have similar hygiene and food safety standards and exchanging food that has already been handled by another individual can thus be a limiting factor. For some individuals, concerns regarding giving up food that they do not perceive as safe, or giving up food very close to its expiration day can be another limiting factor to using the app properly. In contrast, some consumers may consider it a financial gain to keep the food until it is not safe for them to consume, and then give it away, creating concerns regarding app misuse.

Purchasing food from HaFS stakeholders at lower prices can be regarded as a reasonable access to food by individuals with lower income, and it can contribute to food waste prevention. The trade-off with such applications is that certain individuals can make it habit to ‘hunt food offers’ because they have no time or skills to cook a healthy balanced meal, and/or because they become attracted to trying new food, food offers and access to food that would otherwise be too expensive to purchase. This can potentially lead to health-related issues, and ‘hunting food offers’ can become an obsession, which in turn may lead to social problems. An important drawback with the use of online applications is that they exclude access by people who are not technology-savvy, or lack access to appropriate technology. Moreover, the applications are designed to mitigate food waste, which means that food surplus from HaFS may be redistributed to people who are less in need.

### Summarising key findings

**Figure 3.**
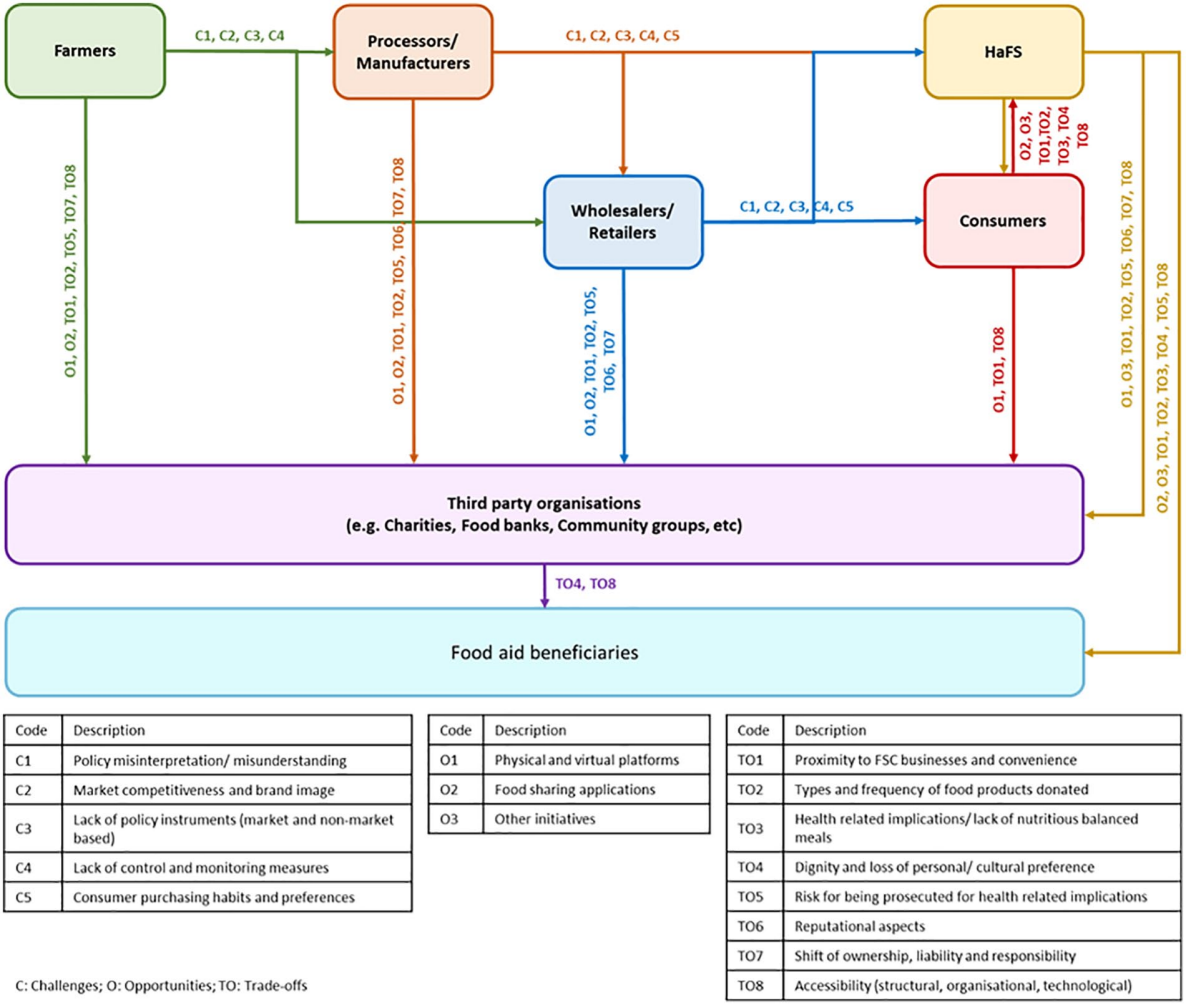
Diagrammatic depiction of the flow of food surplus (arrows) and associated challenges (C), opportunities (O) and trade-offs (TO) in promoting a decrease in avoidable food waste generation.

Figure 3 depicts the challenges, opportunities and trade-offs associated with the potentially avoidable food waste in the form of food surplus, as it flows along the FSC.

Food donor and food aid beneficiaries’ transactions illustrated in Figure 3 are hindered by a number of barriers. A short description of these as identified via our analysis of food regulations, initiatives and strategies, is provided below:

- *C1: Policy misinterpretation/misunderstanding –* stakeholders not confident in understanding the stringency and scope of policy because of wording or mistranslation.
- *C2: Market competitiveness and brand image –* behaviours that arise from competition between stakeholders and from protecting brand image between stakeholders can be counterproductive to increasing food donations.
- *C3: Lack of policy instruments –* some FSC stakeholders are deterred from donating food due to risk of accountability and responsibility for food safety, and because it is financially more attractive to them to maximise profit from selling food products than averting disposal costs through donations.
- *C4: Lack of control and monitoring measures –* good inventory control, such as the supply of just enough product to satisfy consumer demand with no surplus product left unsold is financially unfeasible, and in addition there is a lack of preventive and monitoring measures to avoid overproduction and over-supply that exceeds demand.
- *C5: Consumer purchasing habits and preferences –* consumers drive supply and demand, and, also, the types and aesthetic qualities of food products placed on the market.
- *O1: Physical and virtual platforms –* indirect supply of food surplus to people in need via the operations of non-profit organisations that connect FSC stakeholders at different stages in the FSC with charities and community groups.
- *O2: Food sharing applications –* direct supply of food surplus to people (in need or not) primarily from HaFS.
- *O3: Other initiatives –* direct and indirect supply of food surplus (initiatives from the HaFS sector).
- *TO1: Proximity to FSC businesses and convenience –* distance between donors, charities and/or food aid users may create difficulties for the transport and/or proper handling of food surplus, and inadequate information on such aspects can create inconvenience.
- *TO2: Types and frequency of food products donated* – often the type of food surplus available is not variable enough to help create a nutritious meal, which means that charities and community groups responsible for food distribution directly to people in need have to add the extra ingredients at their own cost; also frequency can be an issue as food surplus may not always available for helping charities/community groups deliver three meals a day every day.
- *TO3: Health-related implications/lack of nutritious balanced meals* – pathways of surplus food distribution that do not guarantee a nutritious balanced meal; there are implications for health when food options available at affordable prices may not be varied enough for a well-balanced diet.
- *TO4: Dignity and loss of personal/cultural preference* – people in need may not feel comfortable receiving aid in certain arrangements, while their choice of food may not be available which means they have to compromise and put aside their preferences.
- *TO5: Risk of being prosecuted for health-related implications –* FSC stakeholders are reluctant to donate food surplus to avoid risk of being accused of health-related implications.
- *TO6: Reputational aspects –* willingness to donate food surplus as quality, freshness and reliability of food products might be compromised, impacting on donors’ reputations.
- *TO7: Shift of ownership, liability and responsibility* – devolution of food product ownership, liability and responsibility for dealing with surplus and damaged food products and EoL management aspects.
- *TO8: Accessibility (structural, organisational, technological)* – refers to organisations that may not have the structural capacity to store, transport or handle food surplus, as well as the inability of FSC stakeholders and/or individuals to engage with the technological means to donate/access food.

Finally the lack of robust data on the types and volume of avoidable food waste and food surplus produced in the UK FSC makes it difficult to identify where avoidable food waste occurs and where interventions are most needed to prevent it (Stenmarck et al., 2016; WRAP, 2018). In turn, this can hinder the implementation of useful policies and instruments to support reduction of avoidable food losses and waste.

## Discussion

Currently, regulatory, structural and organisational aspects cause a restrictive effect on the flow of surplus food redistribution, demotivating businesses from donating high volumes of edible food. Technical, economic, environmental, social and political analysis of the food system is needed to explain observed behaviours, build theories and identify the impact of policy and management actions (Sterman, 2000). Such analyses can be complex, yet they can address important issues in complex systems with multi-causality, stemming from interactions among independent components (Galli et al., 2019; Sterman, 2000; Wu and Huang, 2018). The employment of the CVORR approach for analysing the surplus surplus avoidable food waste management in a broad perspective uncovered a number of challenges, opportunities and trade-offs related to surplus food redistribution. The analysis highlighted multifaceted aspects that need to be scrutinised to enable sustainability in the food system and avoid problems in the face of limited environmental resources and a growing population. These aspects are outlined below.

### Policy reforms

A post-Brexit UK will no longer be required to comply with EU regulations on food, hygiene and consumer information. There are opportunities for policies to be altered or new policies to be formed that may boost food surplus donations and promote productivity in the food system and maximisation of food value recovery, while alleviating food poverty, which is a huge challenge to address even in the UK. Moreover, better management of ‘best before’ and ‘use by’ dates and facilitation of food donations using a flexible traceability system should be introduced. Learning from the successes and failures of models implemented elsewhere (e.g. France, US, Italy) the UK has an opportunity to effectively promote food donation while ensuring food safety. Policy instruments need to be carefully fashioned to streamline an improved control and monitoring process of food supply and demand, and provide the guidelines for food surplus to be exchanged in a timely manner to benefit both the economic and social systems. Collaboration between organisations must be promoted using regulatory instruments, for example, creating a ‘level playing-field’ for businesses, and introducing financial benefits for collaborative research and innovation. Simplification of the health and safety regulations in the UK is essential (Filimonau and Gherbin, 2017).

### Socio-economic reforms

Donating surplus food waste must become more financially attractive to organisations than using alternative methods of treatment (e.g. anaerobic digestion, or composting. A financial incentive can be used to initiate and support preference to surplus food redistribution over food waste management alternatives in the short, medium and long term (Bio by Deloitte, 2014). This will ensure that avoidable food waste generation will be minimised, and that food surplus can reach third-party organisations in a safe and timely manner. This type of intervention (linked to policy reforms) will maximise the value recovered from surplus food. It will roll out benefits for individuals and the local communities that rely on food donations to gain access to food, and ensure that FSC donors extract as much profit from donating their food products in a timely manner as they would if they were selling them (amassed by the incentive(s) and savings gained due to decreasing disposal/EoL management costs). Food banks may relieve the symptoms of food poverty; however, this is not a solution for providing a well-balanced diet and alleviating poverty itself. Concerns associated with the ability of food surplus redistribution initiatives to guarantee a well-balanced diet and propagating further inequalities have been raised, yet more scrutiny on these aspects is required. This points to the fact that, FSC stakeholders and third-party organisations involved in the collection, distribution and handling of food surplus need to work together to guarantee a consistent service to their users and potentially also meet to some extent personal/cultural preferences. Moreover, online applications and technological advancements can be utilised to increase accessibility to a variety of surplus edible foods. For technology-based interventions to succeed in alleviating food poverty, the digital divide between the social classes needs to be resolved. Access to digital technologies (including the internet) are important means to promoting improvements in education, health and wealth (Livingstone et al., 2005).

The conceptual analysis presented in this study showcases the opportunities for intervening in a conventionally structured, unsustainable system that is in urgent need of structural change. An important insight is that stakeholders are inextricably linked to one another and the higher degree of control on stakeholders’ activities is almost always exercised by the stakeholders that come right after them in the FSC. For instance, producers rely heavily on manufacturers and retailers; manufacturers rely on retailers, retailers rely on consumers, and so on. The only exception is consumers who are influenced by a range of factors and stakeholders (both upstream and downstream of the food value chain). Given that food flows downstream on the FSC, it is only logical that this dynamic prevails between the stakeholders involved in the FSC. However, stakeholders operating in the FSC often compete with one other in order to make sure that they best meet their objectives and serve their interests. Competition, however, can stifle progress. For increasing productivity and resource efficiency in the FSC, collaboration between all stakeholders involved in the FSC and innovation are urgently needed. While there is merit in the way: 1) current initiatives promote the recovery and distribution of food surplus to people in need, and 2) food sharing technologies can reduce the amount of food waste generated based on the HaFS-consumer relationships; progress still needs to be made.

There are important benefits from reforming policies on surplus food production, supply and timely management. They can establish a valid ground for creating financial incentives for FSC stakeholders to practise good inventory control and donate food in a timely manner. This can maximise food utilisation and can contribute to developing local food stations, adapting online platforms, and educating the public on safe and effective food waste mitigation strategies. Notwithstanding the important benefits accrued from reforms, and other types of interventions, a good understanding of their potential trade-offs is also required in order to help the UK achieve sustainable circularity in the FSC. This would ensure a successful transition to a productive and resource-efficient FSC system that prevents food waste arising via the improved recovery and redistribution of surplus food. Development of such system not only can result in environmental and economic benefits, but can also help to address food insecurity and poverty in the UK.

## Conclusion

The recovery and redistribution of food surplus can be effective in eliminating avoidable food waste generation and addressing food poverty simultaneously; hence building synergies between food waste reduction and food poverty alleviation. At present, there are many obstacles that hinder progress in salvaging surplus food and redistributing it back into the system for human consumption. There are also many opportunities for promoting sustainability in the FSC, and the UK is on the right track to make the most out of them. Understanding the trade-offs of current initiatives, however, is needed to maximise the benefits gained from these opportunities, and to devise appropriate measures for reinforcing food surplus donations and circularity in the UK FSC. This requires a shift in perspective from seeing stakeholders and their interactions in the food system as isolated components, to seeing them as dynamic elements in the whole food system that interact with the natural, societal, political and economic structures and processes. To that end, the establishment and maintenance of surplus food redistribution activities requires the continuous collaboration of all stakeholders involved in the food value chain, and the implementation of consistent actions across the entire system. A collaboration that is built on mutual benefits, and the desire to promote sustainability in the food system by actively engaging consumers and helping them understand the power of their interests, values, habits and actions in the transition to a sustainable future.

## Notes

1. The Circular Economy Package amends several previous directives: Waste Framework Directive (2008/98/EC); the Landfill Directive (1999/31/EC); the Packaging Waste Directive (94/62/EC); the Directives on end-of-life vehicles (2000/53/EC), on batteries and accumulators and waste batteries and accumulators (2006/66/EC), and on waste electrical and electronic equipment (2012/19/EU).

2. Challenge is defined as something that needs great mental or physical effort in order to be done successfully and therefore tests an individual or group ability to achieve a goal.

3. Opportunity is defined as a situation that makes it possible to do something that an organisation wants to do or has to do, or the possibility of doing something.

4. Trade-off is defined as a situation where something negative is accepted to gain something positive.

5. HaFS sector refers to outlets that provide food and drinks for immediate consumption (e.g. staff catering, healthcare, education, services, quick service restaurants (QSRs) and fast food, restaurants, pubs, hotels and leisure).
